# Using deep learning to identify bladder cancers with *FGFR*‐activating mutations from histology images

**DOI:** 10.1002/cam4.4044

**Published:** 2021-06-10

**Authors:** Constantine S. Velmahos, Marcus Badgeley, Ying‐Chun Lo

**Affiliations:** ^1^ University of Massachusetts Medical School Worcester MA USA; ^2^ Department of Dermatology Mayo Clinic Rochester MN USA; ^3^ Department of Laboratory Medicine and Pathology Mayo Clinic Rochester MN USA

**Keywords:** convolutional neural networks, deep learning, fibroblast growth factor receptors, tumor‐infiltrating lymphocytes, urinary bladder neoplasms

## Abstract

**Background:**

In recent years, the fibroblast growth factor receptor (FGFR) pathway has been proven to be an important therapeutic target in bladder cancer. FGFR‐targeted therapies are effective for patients with *FGFR* mutation, which can be discovered through genetic sequencing. However, genetic sequencing is not commonly performed at diagnosis, whereas a histologic assessment of the tumor is. We aim to computationally extract imaging biomarkers from existing tumor diagnostic slides in order to predict *FGFR* alterations in bladder cancer.

**Methods:**

This study analyzed genomic profiles and H&E‐stained tumor diagnostic slides of bladder cancer cases from The Cancer Genome Atlas (*n* = 418 cases). A convolutional neural network (CNN) identified tumor‐infiltrating lymphocytes (TIL). The percentage of the tissue containing TIL (“TIL percentage”) was then used to predict *FGFR* activation status with a logistic regression model.

**Results:**

This predictive model could proficiently identify patients with any type of *FGFR* gene aberration using the CNN‐based TIL percentage (sensitivity = 0.89, specificity = 0.42, AUROC = 0.76). A similar model which focused on predicting patients with only *FGFR2/FGFR3* mutation was also found to be highly sensitive, but also specific (sensitivity = 0.82, specificity = 0.85, AUROC = 0.86).

**Conclusion:**

TIL percentage is a computationally derived image biomarker from routine tumor histology that can predict whether a tumor has *FGFR* mutations. CNNs and other digital pathology methods may complement genome sequencing and provide earlier screening options for candidates of targeted therapies.

## Graphical abstract

We found that a convolutional neural network, trained on widely accessible tumor histology slides, is highly sensitive at predicting which bladder cancers have a fibroblast growth factor receptor activating mutation. The significance of our finding is that deep learning models, trained on routinely collected histology images, can be a powerful tool to screen for patients who could benefit from targeted therapies.

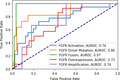

## 1 INTRODUCTION

Bladder cancer is the sixth most common cancer in the United States. Although cure rates are high when malignancy is in situ, survival rates drop significantly if disease is locally invasive or has metastasized.^1^ Therefore, an urgent need exists to develop additional therapeutic options for advanced‐stage bladder cancer and optimize the process of connecting patients to these treatments.

Fibroblast growth factor receptor (*FGFR)* genes regulate cell proliferation, survival, migration, and differentiation.^2^ Furthermore, these genes are commonly mutated in bladder cancer, as abnormal activation of the FGFR pathway is implicated in bladder cancer tumorigenesis and related to metastasis.^3^, ^4^, ^5^ For example, certain mutations, such as that of *FGFR3*, are found in approximately 75% of low‐grade papillary bladder urothelial carcinoma, while overexpression of the FGFR3 protein is associated with high‐grade, aggressive disease.^6^, ^7^ As some studies have discovered various *FGFR* mutations in up to 60% of all urothelial carcinoma and *FGFR3* mutations in 15% of metastatic urothelial carcinoma, new bladder cancer therapies have begun targeting patients with *FGFR*‐specific mutations.^8^ Erdafitinib, an FDA‐approved, second‐line drug, inhibits the FGFR pathway and has proven to be particularly potent for patients with high‐grade bladder cancer. In contrast to current second‐line drug therapies, where patients typically face 5‐year survival rates of 15%, Erdafitinib achieves significantly higher survival rates than its predecessors by being a potent tyrosine kinase inhibitor of FGFR 1–4.^9^ Thus, Erdafitinib and other FGFR targeting drugs would be appealing alternative therapeutic options for patients with *FGFR* mutations who do not respond to first‐line treatments. However, systems to rapidly and accurately identify these metastatic bladder cancer patients with *FGFR* mutations are lacking.

Currently, targeted genome sequencing is the primary method of determining an individual's tumor genetic profile. While access to sequencing is improving, it is not a universal practice for all cancer treatments due to financial restrictions and resource limitations. This lack of sequencing also affects clinical trial enrollment for targeted agents. It is difficult, inefficient, and expensive to sequence a large cohort of patients in order to determine which ones have qualifying *FGFR2/3* mutations and could be candidates for FGFR‐inhibiting drug therapies. As a result, widespread access to potentially beneficial drugs may be often delayed and trial enrollment time may be prolonged.

This study sought to overcome the aforementioned issue by addressing two major needs related to personalized therapy and clinical trials. First, we sought to identify an easily accessible surrogate biomarker correlated with a patient's *FGFR2/3* mutational status. Second, we aimed to develop a high‐throughput method, which utilizes the surrogate marker, to rapidly screen for patients that may be eligible for targeted medications.

Tumor‐infiltrating lymphocytes (TIL) have been shown to be an important component within a tumor microenvironment and are commonly visible in tumor pathology slides.^10^ The presence of TIL is positively correlated to overall survival in bladder cancer and inversely correlated to *FGFR2/3*‐mutated genetic status.^11^ Therefore, low TIL presence in a patient's tumor microenvironment may serve as a surrogate biomarker of *FGFR2/3* genetic activation.

We hypothesized that by utilizing deep learning models, known as convolutional neural networks (CNN), we can computationally analyze pathology slides, determine the presence of TIL, map patient *FGFR* gene mutation status to the presence of TIL, and use CNN to rapidly and efficiently screen large populations of cancer patients for possible *FGFR* gene alteration, in order to find qualifying drug‐recipient candidates.

## 2 METHODS

This study includes all bladder cancer cases of The Cancer Genome Atlas (TCGA), a publicly available dataset, curated jointly by the National Cancer Institute and National Human Genome Research Institute. Since 2006, over 20,000 primary cancer and matched normal cases, composed of 33 cancer types, have been sequenced and recorded in the dataset. The dataset consists of genomic, epigenomic, transcriptomic, proteomic, and histological data.

### 2.1 Determining TIL percentage in bladder cancer cases with diagnostic whole slide images

Of 418 bladder cancer cases, 386 cases had accompanying digitized, H&E stained whole slide images (WSI) of diagnostic tumor biopsies. Of the cases with accompanying WSI, 290 had gene mutation and RNA‐seq data available to assess *FGFR* mutation status, gene fusion variants, and RNA expression status (Figure S1).

To quantify the amount of TIL in a WSI, we utilized a TIL percentage estimation CNN developed by Saltz et al.^12^ In brief, the TIL percentage CNN was a semi‐supervised CNN. The initial pre‐training of the CNN was performed by an unsupervised convolutional autoencoder. The TIL percentage CNN was then built on top of the unsupervised convolutional autoencoder, and used pathologist TIL‐labeled images for training and optimization. Further information on the CNN architecture and validation of the deep learning model can be found in Saltz et al.’s peer‐reviewed manuscript.

Each WSI was divided into 50 × 50 μm^2^ square tiles (“patches”), corresponding to 20× magnification and 10^4^ patches per image. Then, the CNN was trained to detect the presence of TIL in one image patch. For each slide, its “TIL percentage” was assigned by dividing the total number of patches with TIL present (determined by the CNN) by the total number of patches. TIL percentage values were then computed for each bladder cancer patient. A Wilcoxon rank‐sum statistic was utilized to statistically compare TIL percentage distributions between different subpopulations. A *p*‐value of less than 0.05 was considered to be significant.

### 2.2 Comparison of TIL percentage estimation by CNN and direct pathological assessment

A direct pathological assessment of TIL is conducted. The same set of digital images were evaluated by a practicing pathologist for TIL, blinded to the CNN results and molecular correlation. TIL evaluation was scored on a 0 to 3+ semi‐quantitative scale based on the quantity of infiltrated lymphocytes in the tumor area. While 0 indicates the absence of TIL, 1+ to 3+ were assigned when mild, moderate, or marked TIL were identified. Stability of the predictive capabilities of TIL percentage and pathologist scoring were analyzed via 5‐fold cross‐validation logistic regression model.

### 2.3 Determination of driver gene mutation and FGFR activation status

Three hundred twenty‐four genes, which are routinely screened as part of tumor mutation gene panels, such as in the Foundation Medicine FoundationOne CDx genomic test, were included in the analysis.^13^ Gene mutation status of all 324 genes were acquired from TCGA Genome Data Commons Data Portal.^14^ Genes with lower than 10% mutation prevalence in the analyzed bladder cancer subset were excluded from subsequent predictive model development to avoid scenarios of extreme class imbalance.

Furthermore, we included several types of *FGFR* aberrations in our analysis, such as *FGFR* fusion, overexpression, or amplification. To identify cases with *FGFR* fusions, we queried the Fusion Gene annotation DataBase for all bladder cancer cases with *FGFR2* or *FGFR3* fusion. To identify cases with *FGFR2*/*FGFR3* overexpression or amplification, we acquired normalized gene expression RNA‐seq and copy number datasets of bladder cancer cases from the University of California, Santa Cruz (UCSC) Genome Browser.^15^ We refer to this collective of *FGFR2/FGFR3* mutation, fusion, overexpression or amplification, as “*FGFR* activating mutation” in our manuscript. *FGFR* gene “overexpression” was defined as an *FGFR* RNA aberration, where significantly more RNA copies of the *FGFR* gene were found in a patient, in comparison to the control group. *FGFR* gene “amplification” refers to an increased number of copies of the gene found in a patient's genome.

### 2.4 Prediction of gene mutation status using TIL percentage

A univariate logistic regression model was developed to predict the presence of each gene mutation using TIL percentage as input. 80% (*n* = 232) of the cases were selected randomly and utilized as a development set to train the machine learning predictor, while the remaining 20% (*n* = 58) were used as a test set to measure the model's performance. The model was assessed using area under the ROC (AUROC).

## 3 RESULTS

### 3.1 Distribution of TIL percentage and prevalence of genetic alterations in bladder cancer subset

The demographic characteristics of this bladder cancer cohort are summarized in Table 1. Out of all the bladder cancer cases, 32% were found to have any kind of *FGFR* activating mutation, of which 24% was *FGFR2* or *FGFR*3 gene overexpression (>1 standard deviation across bladder cancer subset), 19% was *FGFR2* or *FGFR3* driver mutation, 5% was *FGFR2* or *FGFR3* gene amplification, and 3% was *FGFR2* or *FGFR3* fusion.

**TABLE 1:** Patient population characteristics

Population characteristics	Bladder cancer patients (*n* = 290)
Median TIL percentage (%)	5.34 (range: 0.02–44.68)
Age at pathological diagnosis (years)	68 (range: 34–90)
Gender	
Male	220 (76%)
Female	70 (24%)
Race	
Asian	41 (14%)
Black or African American	20 (7%)
Other	10 (3%)
White	219 (76%)
AJCC pathologic staging	
Stage I	1 (1%)
Stage II	95 (33%)
Stage III	100 (34%)
Stage IV	94 (32%)
Histological grade	
High grade	269 (93%)
Low grade	21 (7%)
Types of *FGFR* mutation	
*FGFR2*/*FGFR3* gene overexpression	70 (24%)
*FGFR2*/*FGFR3* driver mutation	55 (19%)
*FGFR2*/*FGFR3* gene amplification	15 (5%)
*FGFR2*/*FGFR3* fusion	9 (3%)
Any kind of *FGFR* activating mutation	93 (32%)

Abbreviations: AJCC, American Joint Committee on Cancer; TCGA, The Cancer Genome Atlas; TIL, tumor‐infiltrating lymphocytes.

**FIGURE 1:**
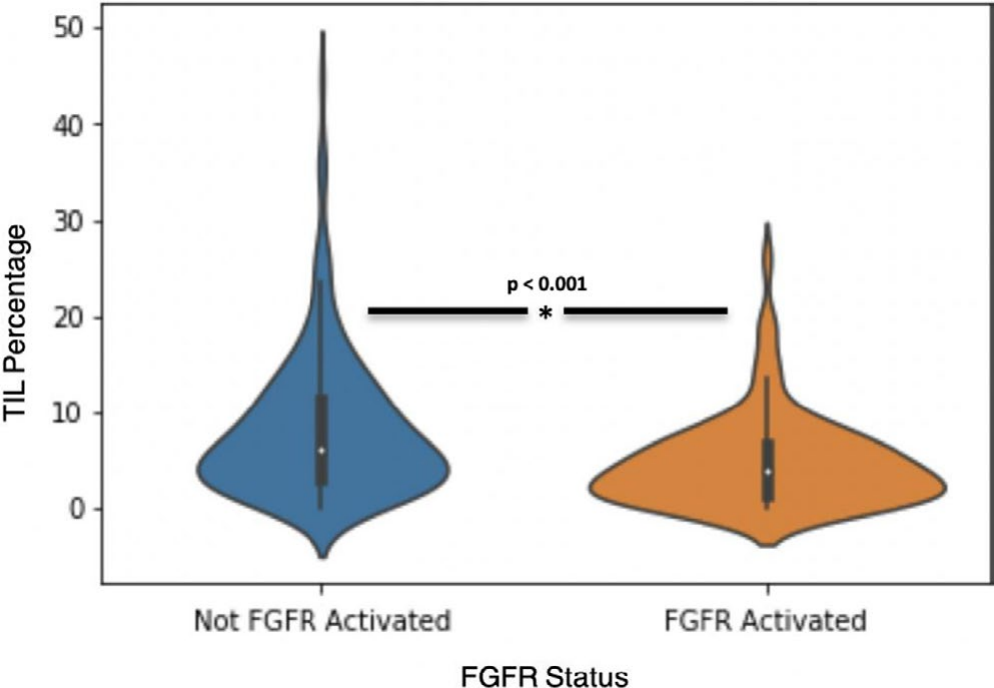
Distribution of TIL percentage stratified by FGFR activation status

**FIGURE 2:**
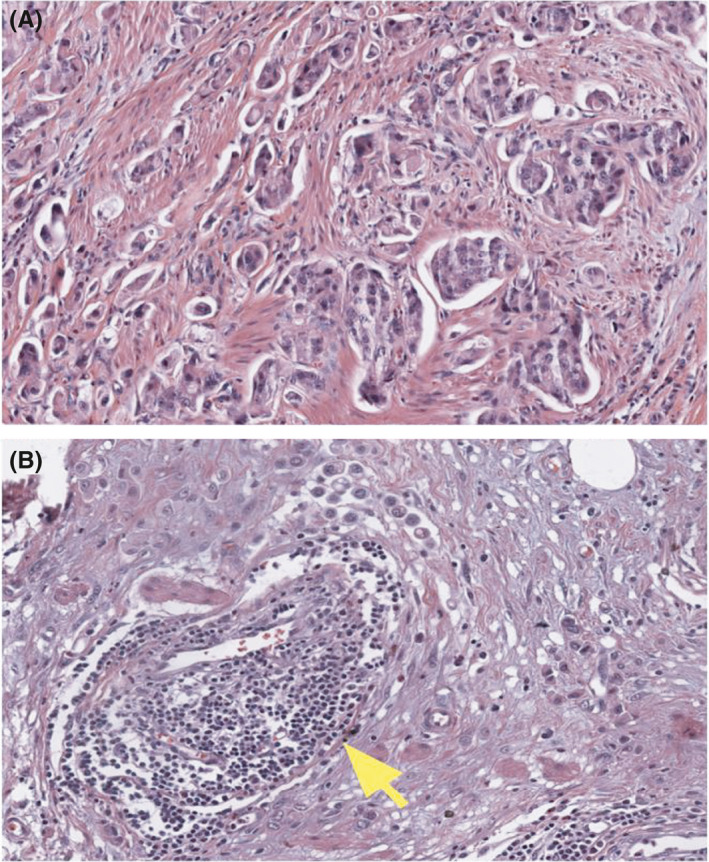
Representative histology images of bladder urothelial carcinomas: (A) A high‐grade urothelial carcinoma with *FGFR2* overexpression by RNAseq shows tumor infiltrating the stroma with minimal TIL; (B) A similar high‐grade urothelial carcinoma with no *FGFR* alteration shows stromal reaction, occasional TIL, and tumor‐associated lymphoid aggregates (arrow)

Utilizing CNN, we computed the overall median image‐based TIL percentage to be 5.34% (range: 0.02–44.68) in this bladder cancer population (Table 1). The distribution of image‐based TIL percentage of FGFR activated and FGFR wild‐type bladder cancer is shown in Figure 1; FGFR activated bladder cancer cases had more TIL compared to their FGFR wild‐type counterparts (*p* < 0.001). Representative H&E‐stained images from an FGFR activated and FGFR wild‐type bladder cancer case are presented in Figure 2.

### 3.2 Predictive performance of logistic regression models using TIL percentage

**FIGURE 3:**
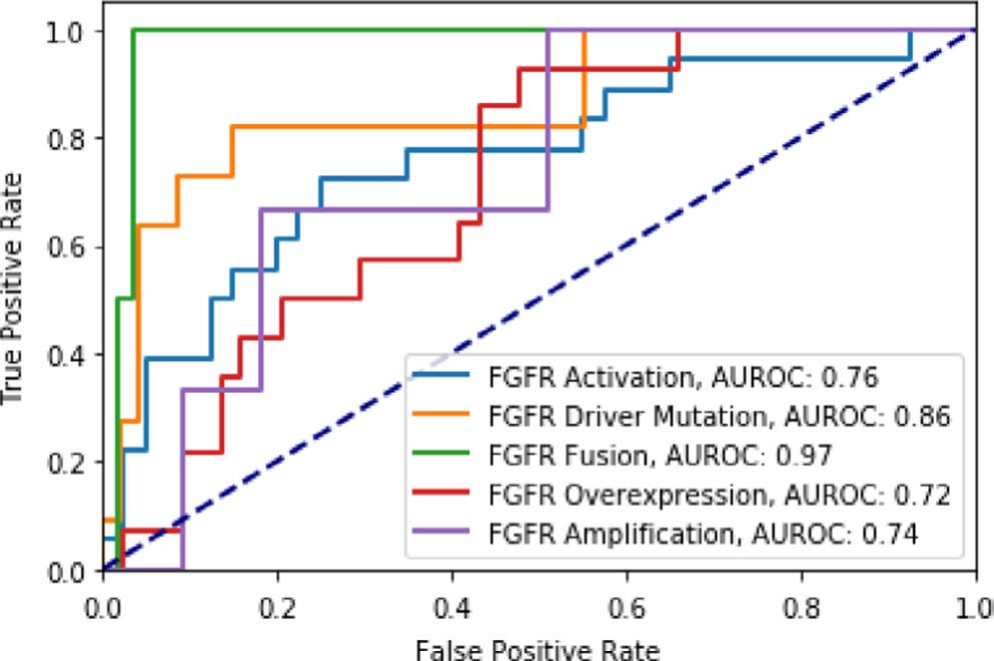
Model performance for the prediction of various types of *FGFR* activating mutation using TIL percentage

Within the cohort of 290 bladder cancer cases included in the analysis, 11 genes with mutation prevalence greater than 10% were included for predictive modeling (Table 2). A logistic regression model, using TIL percentage, predicted *FGFR2/3* driver mutation, fusion, gene overexpression, amplification, as well as any type of *FGFR* activating mutation with AUC of 0.86, 0.97, 0.72, 0.74, and 0.76, respectively. The ROC curves for all these *FGFR* predictions are reported in Figure 3. As the goal of the study was to create a screening tool for genetic mutation, the high‐sensitivity operating point for *FGFR2/3* driver mutation was identified, with a sensitivity of 0.82, specificity of 0.85, positive predictive value (PPV) of 0.56, and negative predictive value (NPV) of 0.95. For any kind of *FGFR* activating mutation, the sensitivity, specificity, PPV, and NPV were determined to be 0.89, 0.42, 0.43, and 0.9, respectively.

**TABLE 2:** Prediction performance for mutated genes with a prevalence of more than 10%

Gene mutation	Logistic regression AUC	Positive cases (*n* = 290)
*FGFR3*	0.85	53 (18%)
*TP53*	0.72	139 (48%)
*RB1*	0.57	75 (26%)
*PIK3CA*	0.55	60 (21%)
*CREBBP*	0.48	38 (13%)
*CDKN2A*	0.46	112 (39%)
*CCND1*	0.44	36 (12%)
*CDKN2B*	0.37	95 (33%)
*RAF1*	0.36	32 (11%)
*E2F3*	0.30	49 (17%)
*ERBB2*	0.21	37 (13%)
*FGFR2/FGFR3* mutation	0.74	55 (19%)

Abbreviation: AUC, area under the curve.

Predictive AUC values and ROC curves for all remaining genetic alterations are included in Table 2 and Figure S2, respectively. Other than *FGFR*, TIL percentage was not predictive of other genetic aberrations.

### 3.3 Pathologist assessment of TIL in comparison to TIL percentage as a predictor

**FIGURE 4:**
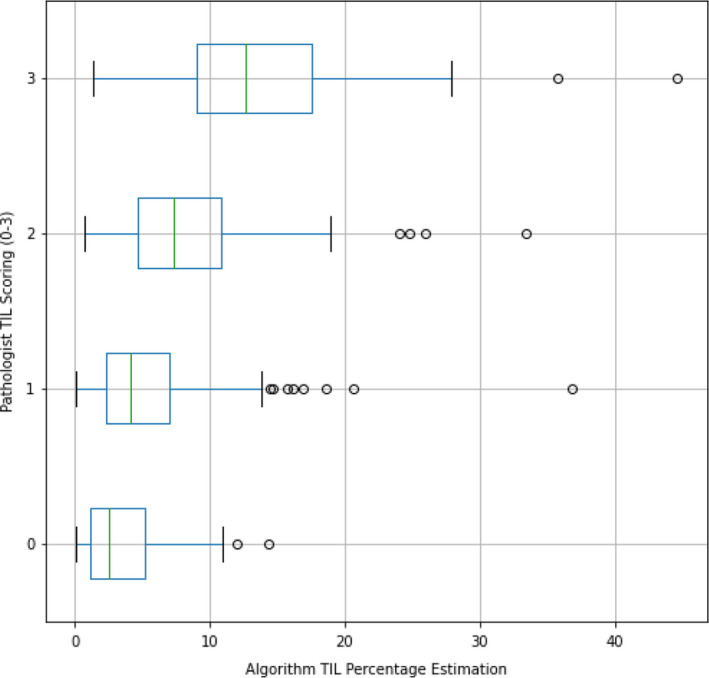
Direct pathologist scoring and TIL percentage for each TCGA bladder cancer image

**FIGURE 5:**
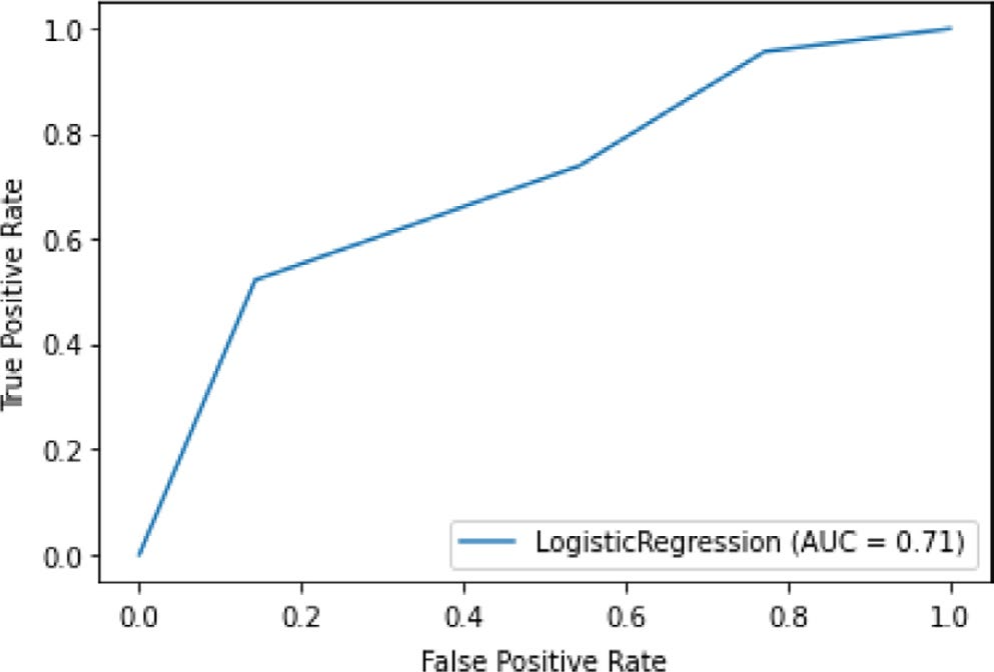
Model performance for the prediction of *FGFR* activating mutation using direct pathologist scoring on the overall test set

**FIGURE 6:**
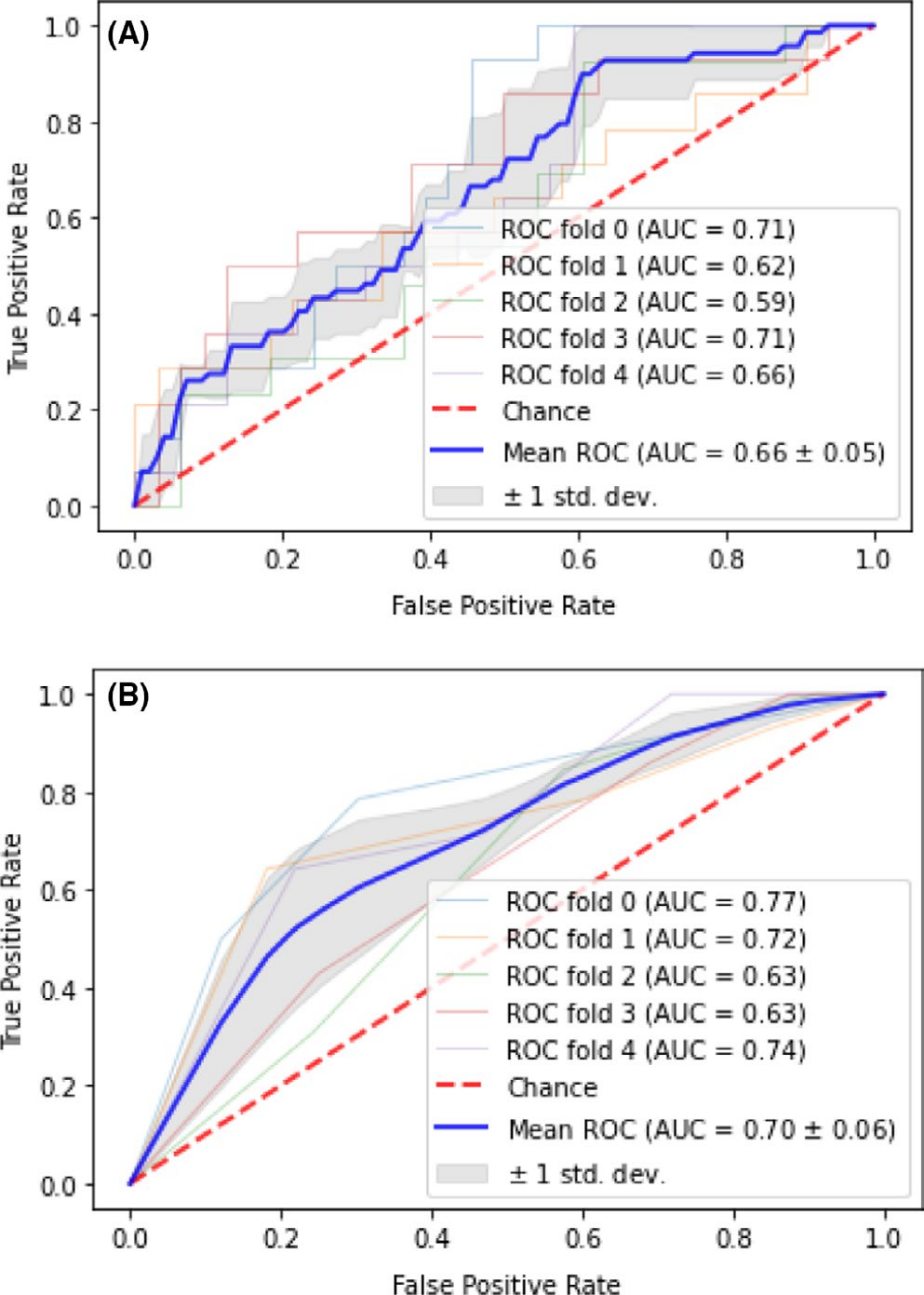
Cross‐validated model performance for the prediction of *FGFR* activating mutations utilizing (A) TIL percentage and (B) Direct pathologist scoring

TIL percentage and pathologist TIL scoring were reported for each image in Figure 4. On the overall test set, TIL percentage (AUC = 0.76) was a stronger predictor of FGFR activation than pathologist TIL scoring (AUC = 0.71), which contrasted with the cross‐validation models, where pathologist TIL scoring (AUC = 0.70 + 0.06) performed better than TIL percentage (AUC = 0.66 + 0.05) (Figures 5 and 6).

## 4 DISCUSSION

In the era of precision medicine, targeted therapies for patients with specific molecular alterations have become the focus of drug development efforts and oncology clinical care. Across different tumor subtypes, as many as 26 genomic biomarkers have received FDA approval as companion diagnostics for targeted oncology therapy.^16^ Specifically in bladder cancer, these FDA‐approved, companion diagnostic biomarkers are *FGFR3* mutation and *FGFR2*/*3* fusion. Despite the growing availability of beneficial, FGFR‐inhibiting therapies, the widespread applicability of such treatments may be limited or delayed by current genomic screening practices. A 2018 national survey revealed that only one in five oncologists reported frequent usage of targeted panels at the time of initial cancer diagnosis, as, indeed, oncologists often resort to targeted therapy only in later stages of disease.^17^

As such, the genomic profile of the majority of cancer patients at initial diagnosis remains unknown, barring them from inclusion in clinical trials and from receiving potentially beneficial, targeted therapies in the early stages of disease. Given that genetic profiling is expensive and time‐intensive, it can be unrealistic to sequence large populations. Minimally invasive methods to screen cancer patients early have also been developed, such as those utilizing circulating tumor DNA (ctDNA). However, ctDNA methods are barred from extensive clinical use due to many of the same issues that preclude prevalent genetic profiling, namely cost and complexity.^18^ So, there is great utility in determining efficient, widespread, and feasible early stratification methods to complement tumor genomic sequencing or other sources of minimally invasive screening. As a result, patients would not only be connected with potential life‐saving therapies, but also a load of molecular and genetic assays ordered and utilized could be lightened significantly. In this study, we seek to identify such bladder cancer patients through a low‐cost and minimal‐risk method using existing tumor diagnostic histology images.

We showed that a computationally derived imaging biomarker for TIL presence, TIL percentage, can predict *FGFR2/3* mutation and, more broadly, *FGFR* activating mutation in bladder cancer. Importantly, TIL percentage is calculated directly from patient tumor slides taken at the time of initial diagnosis. In addition to the observed predictive value of TIL in our machine learning experiments, the inverse relationship between TIL presence and *FGFR* activating mutation is corroborated by multiple prior molecular studies, which found that the alteration of FGFR pathways is implicated in suppression of lymphocyte infiltration in urothelial carcinoma.^19^, ^20^ Furthermore, TIL percentage was not a good predictor of non‐FGFR mutations, suggesting that either TIL or the histopathological‐determined TIL percentage value are sub‐optimal surrogate markers for genetic alterations in RAF1, E2F3, etc. (Figure S2). In cases of urothelial cancer, causal relationships between TIL and most of these genes remain sparse in the current literature, however, one study found that RAF1 fusions were associated with low TIL presence in melanoma cases.^21^

**FIGURE 7:**
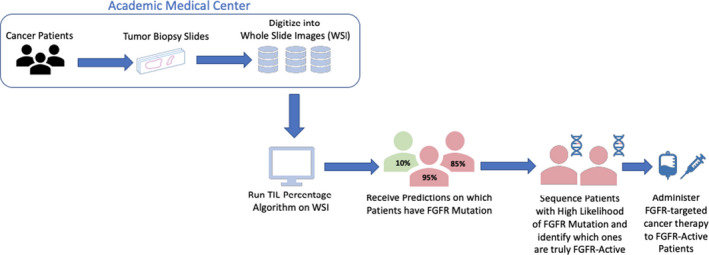
Schematic of *FGFR* activating mutation screening workflow using imaging‐based prediction algorithm

We found that a TIL percentage‐based logistic regression prediction model had excellent performance for the detection of *FGFR* driver mutations, with an AUC of 0.86 and a well‐balanced operating point with a sensitivity of 0.82 and specificity of 0.85. The performance for *FGFR* driver mutation detection was also particularly encouraging: TIL percentage could be utilized to screen for rare, *FGFR* activated cases in large patient populations, where genetically sequencing all cases would not be efficient nor practical. We propose a workflow in Figure 7 that would incorporate our model along with confirmatory sequencing to rapidly connect such patients with targeted therapy. This methodology requires digitized, H&E stained WSI of diagnostic tumor biopsies followed by neural network techniques, which are complex, and often not intuitive to individuals unfamiliar with this technology. However, the digitization of pathology slides has been rapidly adopted as common practice in many medical centers and we believe that the implementation of such a workflow is feasible in the near future. Designed in a user‐friendly manner, platforms utilizing powerful CNN’s could be applied by physicians or clinical trial coordinators to parse through large populations of potential drug therapy candidates.

For example, our high negative predictive value for *FGFR2/3* mutation, as well as any kind of *FGFR* mutation, could be useful in the clinical setting. It may allow clinicians to efficiently and successfully parse out patients whose tumor likely does not bear *FGFR* aberrant mutations, without the need to perform expensive tumor genomic testing for each patient. This is particularly valuable in resource‐limited practices to avoid low‐yield molecular testing. Though our method can help identify a concentrated patient population, enriched in *FGFR* alterations, this morphology‐based alternative platform can never completely replace tumor genomic testing. Our method could be implemented to stratify patients for FGFR inhibitor eligibility, yet, the ultimate identification of which patients will receive FGFR inhibitor treatment necessitates the use of genetic testing. We additionally analyzed precision‐recall curves of our various mutations and observed similar results, suggesting that, indeed, our platform could be utilized in categorizing patients into FGFR‐activated and FGFR non‐activated groups.

The difference in the presence of TIL between patients with *FGFR* wild‐type and *FGFR*‐mutated bladder cancer can be subtle, as shown in Figure 1. It would be extremely challenging for pathologists to consistently identify minute differences in TIL percentage through mere observation. However, digital image analysis with CNN provides a consistent and rapid manner to quantitatively estimate the TIL percentage on any given pathology specimen, making CNN a reliable, useful, and complementary tool for pathologists to use when identifying bladder cancer patients with *FGFR* activating mutation. Additionally, CNN could provide insight on patient tumor genetic profiles, as TIL percentage was able to predict a wide variation of genetic variations found in FGFR‐active patients, such as the presence of copy number variants, or unusual RNA overexpression.

In multiple studies, CNN has proved to be particularly efficient and accurate at classifying histology slides and extracting key information for the diagnosis and treatment of certain diseases, such as melanoma, occasionally outperforming physician predictions.^22^, ^23^, ^24^ Similarly, previous studies have used pan‐cancer approaches in correlating CNN‐extracted variables with some genotype‐phenotype correlations, gene expression profiles, and localization of immune cells.^25^, ^26^, ^27^ However, current literature is lacking in the delicate prediction of *FGFR* genetic alterations. As the *FGFR* gene is a well‐known target for urothelial cancer‐targeted therapies, our study uniquely provided a method, utilizing CNN, for the prediction of this specific, yet important gene. The literature suggests that CNN may be the key tool to optimize the patient screening process of clinical trials, through the ability to process vast amounts of information efficiently and in a high‐throughput manner.^28^, ^29^ Beyond TIL percentage, CNN holds the ability to derive many other histological metrics, such as tumor diameter, tumor area, and mucosal length. These variables could be rapidly analyzed through CNN to discover novel surrogate markers for other genes in different tumor types.

Our study is limited by certain factors, such as the generalization of the CNN used. Extraction of TIL percentage was performed by a CNN that was trained specifically on TCGA data. While TCGA is built on samples from multiple institutions, this CNN may not generalize as well to other institutional data. For example, the TCGA dataset consists of patients’ primary tumor samples, which would certainly have varying levels of lymphocyte infiltration when compared to specimens from metastatic lesions. In future experiments, we intend to utilize metastatic tumor samples to train the model on varying levels of lymphocyte infiltration, which would allow for additional insight on FGFR activation prediction using computer‐analyzed WSI. Since bladder urothelial carcinoma is not recommended for routine profiling by molecular testing under the current National Comprehensive Cancer Network clinical practice guidelines, we were unable to identify a well digitalized and molecularly annotated dataset to perform an external validation. We plan to acquire an external institutional dataset to re‐train our CNN, observe its generalizability, and fine‐tune our methodology to validate the relationship between FGFR activation and TIL presence. This validation would serve to develop a more robust machine learning algorithm to predict FGFR activation from TIL image metrics. We hope that this study illustrates the potential for utilizing tumor‐morphology CNN analysis to better patient care and will encourage institutions to begin collecting such datasets.

In conclusion, our study demonstrated a powerful potential screening tool for bladder cancer patients with *FGFR* mutation. Utilizing a histologically derived metric, TIL percentage, we were able to generate a predictive tool that could confidently screen for *FGFR2/3* gene mutation, as well as other *FGFR* aberrations, in a large population of bladder cancer patients. In the age of big data and precision medicine, CNN and other emerging digital pathology methods, may serve as the optimal tools to efficiently screen vast populations and match them to potentially life‐saving, beneficial, targeted therapies.

## CONFLICT OF INTERESTS

At the time that the research took place, Constantine S. Velmahos and Marcus Badgeley were full‐time employees, while Ying‐Chun Lo was an external consultant, at a for‐profit organization, named *nference* that specializes in the biomedical artificial intelligence space. This organization has collaborations with Janssen, the pharmaceutical subsidiary of Johnson & Johnson, and producer of the drug Erdafitinib, which is mentioned but not commercially promoted in this manuscript. All authors declare no more competing interests.

## AUTHORS’ CONTRIBUTIONS

MB and CSV conceived of the presented study's idea and performed the analysis. YC performed the pathology review. CSV wrote the manuscript. CSV, MB, and YCL reviewed and revised the manuscript.

## Supporting information

Fig S1

Fig S2

## Data Availability

The data that support the findings of this study are openly available in The Cancer Genome Atlas GDC Portal at https://portal.gdc.cancer.gov/.
