# Supplementary figures and images for: Using deep learning to identify bladder cancers with FGFR‐activating mutations from histology images
Source: Cancer Med. 2021 Jun 10;10(14):4805–13. doi: 10.1002/cam4.4044 (PMC8290253; doi:10.1002/cam4.4044)

Supplementary Figure 1 - Patient inclusion & exclusion flow chart


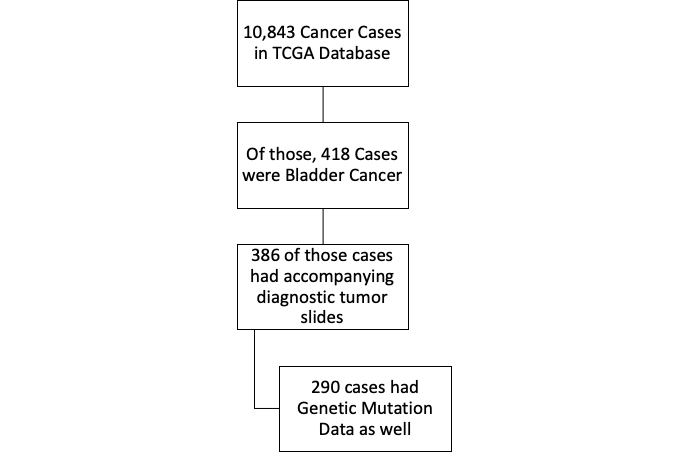

Supplement: Supplementary file 1 — Fig S1 [file CAM4-10-4805-s002.docx]
