# Supplementary material for: Using deep learning to identify bladder cancers with FGFR‐activating mutations from histology images
Source: Cancer Med. 2021 Jun 10;10(14):4805–13. doi: 10.1002/cam4.4044 (PMC8290253; doi:10.1002/cam4.4044)

Supplementary Figure 2 - ROC analysis of genes with greater than 10% mutation prevalence, which are A) *CCND1*, B) *CDKN2A*, C) *CDKN2B*, D) *CREBBP*, E) *E2F3*, F) *ERBB2*, G) *PIK3CA*, H) *RAF1*, I) *RB1*, and J) *TP53*.


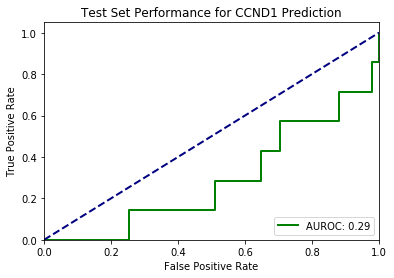


A

B


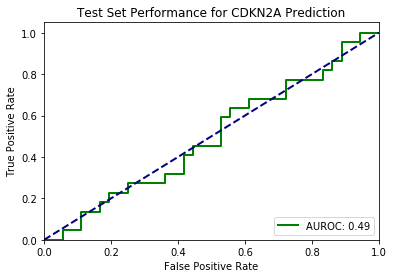


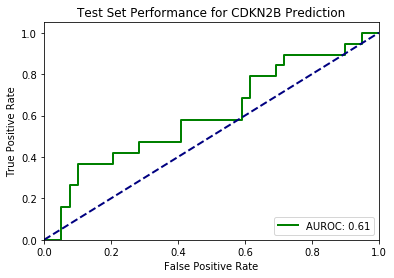


D

C


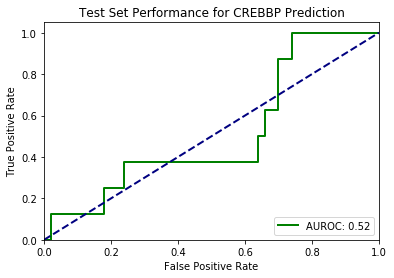


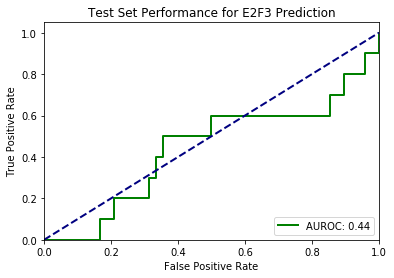


E


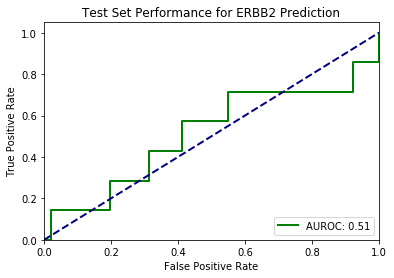


F


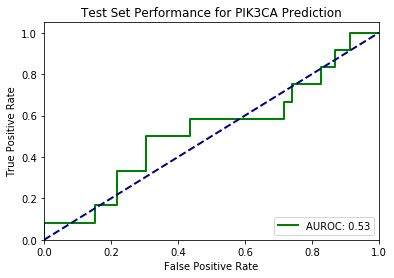


H

G


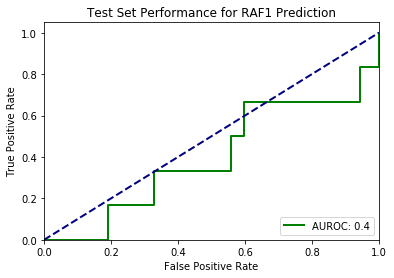


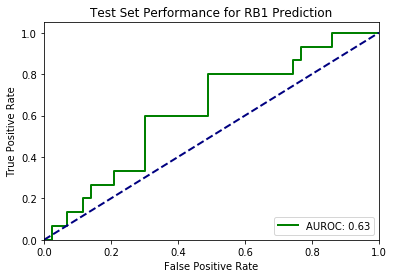


J

I


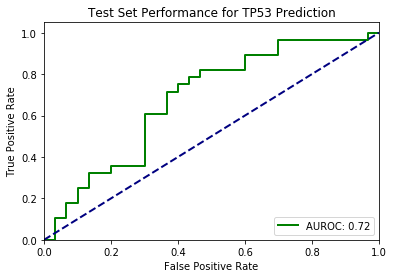

Supplement: Supplementary file 2 — Fig S2 [file CAM4-10-4805-s001.docx]
